# Extensive remodeling of the *Pseudomonas syringae* pv. *avellanae* type III secretome associated with two independent host shifts onto hazelnut

**DOI:** 10.1186/1471-2180-12-141

**Published:** 2012-07-16

**Authors:** Heath E O’Brien, Shalabh Thakur, Yunchen Gong, Pauline Fung, Jianfeng Zhang, Lijie Yuan, Pauline W Wang, Choseung Yong, Marco Scortichini, David S Guttman

**Affiliations:** 1 Department of Cell and Systems Biology, University of Toronto, 25 Willcocks St., Toronto, ON, M5S 3B2, Canada; 2 Center for the Analysis of Genome Evolution & Function, University of Toronto, 25 Willcocks St., Toronto, ON, M5S 3B2, Canada; 3 C.R.A.- Fruit Crops Research Centre, Via di Fioranello, 52; I-00134, Rome, Italy

**Keywords:** Effector, Host specificity, Molecular dating

## Abstract

**Background:**

Hazelnut (*Corylus avellana*) decline disease in Greece and Italy is caused by the convergent evolution of two distantly related lineages of *Pseudomonas syringae* pv. *avellanae* (*Pav*). We sequenced the genomes of three *Pav* isolates to determine if their convergent virulence phenotype had a common genetic basis due to either genetic exchange between lineages or parallel evolution.

**Results:**

We found little evidence for horizontal transfer (recombination) of genes between *Pav* lineages, but two large genomic islands (GIs) have been recently acquired by one of the lineages. Evolutionary analyses of the genes encoding type III secreted effectors (T3SEs) that are translocated into host cells and are important for both suppressing and eliciting defense responses show that the two *Pav* lineages have dramatically different T3SE profiles, with only two shared putatively functional T3SEs. One *Pav* lineage has undergone unprecedented secretome remodeling, including the acquisition of eleven new T3SEs and the loss or pseudogenization of 15, including five of the six core T3SE families that are present in the other *Pav* lineage. Molecular dating indicates that divergence within both of the *Pav* lineages predates their observation in the field. This suggest that both *Pav* lineages have been cryptically infecting hazelnut trees or wild relatives for many years, and that the emergence of hazelnut decline in the 1970s may have been due to changes in agricultural practice.

**Conclusions:**

These data show that divergent lineages of *P. syringae* can converge on identical disease etiology on the same host plant using different virulence mechanisms and that dramatic shifts in the arsenal of T3SEs can accompany disease emergence.

## Background

*Pseudomonas syringae* is a Gram-negative plant pathogen that causes a spectrum of speck, spot and canker diseases on a range of plant hosts. It is divided into approximately 50 pathovars (pathogenic varieties) that are specialized for particular host plants and are generally unable to cause disease on other species. Multilocus sequence analysis (MLSA) has shown that many pathovars correspond to distinct evolutionary (monophyletic) lineages [1,2]. A notable exception to this pattern is *P. syringae* pv. *avellanae* (*Pav*), where two distantly related lineages within *P. syringae* have converged upon a common disease phenotype on hazelnut (*Corylus avellana*) plantations in Greece and Italy. *Pav*-associated hazelnut decline characterized by wilting of branches and trunk cankers was first observed in Greece and Italy in the mid 1970s, though the disease was not formally described in Italy until the 1990s [3]. MLSA has shown that all isolates from Greece form a distinct lineage related to pathogens of kiwifruit (*P. syringae* pv. *actinidiae*; *Pan*[4], a.k.a. *Psa*[5]) and plum (*P. syringae* pv. *morsprunorum*; *Pmp*) in phylogroup 1. This phylogroup also includes a large number of pathogens of herbaceous plants, including the well-studied *P. syringae* pv. *tomato* strain *Pto* DC3000. In contrast, Italian isolates collected during outbreaks in the 1990s cluster together in phylogroup 2, along with pathogens of peas, cereals, and other plants, including the well-studied *P. syringae* pv. *syringae* strain *Psy* B728a. More recent outbreaks of hazelnut decline in Italy from 2002–2004 were caused by *Pav* that phylogenetically clusters with the Greek isolates in phylogroup 1.

In order to determine the genetic changes accompanying the evolution of hazelnut pathogenesis in these two independent lineages, we obtained draft whole genome sequences for the earliest isolate of the hazelnut decline pathogen, *Pav* BP631, a phylogroup 1 strain isolated from Drama, Greece in 1976 and for *Pav* Ve013 and *Pav* Ve037, two strains isolated in Rome, Italy in the early 1990s. The latter two strains represent the extremes of genetic diversity observed in phylogroup 2 *Pav* strains as determined by the MLSA analysis of Wang *et al.*[6]. This MLSA analysis indicates that *Pav* Ve037 clusters with pea pathogens (*P. syringae* pv. *pisi*; *Ppi*) while the other strains group with pathogens of beets (*P. syringae* pv. *aptata*; *Ptt*) and barley (*P. syringae* pv. *japonica*; *Pja*) although with very weak phylogenetic support.

We compared these three draft genome sequences to 27 other complete or draft *P. syringae* genome sequences representing 16 pathovars, including seven phylogroup 1 strains and six phylogroup 2 strains [4,7-17]. We performed ortholog analysis to identify instances of horizontal gene transfer between the two independent *Pav* lineages and looked in detail at the evolutionary histories of a number of candidate pathogenicity genes, including the type III secreted effectors (T3SEs) that are translocated into host cells and are important for both suppressing and eliciting defense responses. We show that the two lineages have dramatically different T3SE profiles and that *Pav* BP631 has undergone extensive secretome remodeling.

## Results

### Genome sequencing and assembly

**Figure 1:**
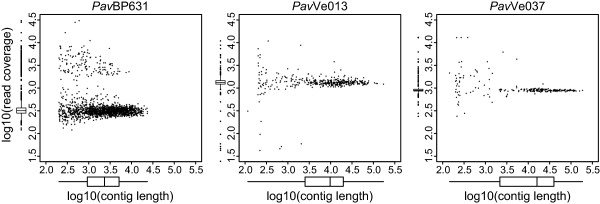
**Coverage plots for contigs generated for each*Pav*strain.** Read coverage vs. contig length, plotted on log scales. Box and whisker boxes indicate median, quartiles, and range for each strain, with values more than 2.5 times the interquartile range above or below the median plotted as points. Data were plotted using the car package in R [18,19].

43 million read pairs were generated from the *Pav* BP631 paired-end library, while the *Pav* Ve013 and *Pav* Ve037 paired-end libraries produced 59 million and 35 million read pairs respectively (Table 1). The 82 bp reads for the latter two strains resulted in considerably longer contigs (N50s of 31 kb and 61 kb) than the 38 bp *Pav* BP631 reads (N50 of 6.4 kb). The read depth of the contigs was very uniform for *Pav* Ve013 and *Pav* Ve037, with almost all the contigs centered around a depth of 1000X (Figure 1). In contrast, the majority of the *Pav* BP631 contigs were centered around a depth of 300x, but there were also a large number with depth in the thousands, including some up to almost 10,000 bp in length. These high-coverage contigs indicate that this strain harbors one or more multi-copy plasmids.

**Table 1:** Genome statistics for strains sequenced in this study

**Strain**	**Cluster # **^**1**^	**Contig #**	**Contig N50**	**Scaffold #**	**Scaffold N50**	**Genome size**	**ORFs**
*Pav*BP631	43 M^2^ 38 bp PE	1,613	6,420	297	79,231	6,628,588	4816
	38 M 38 bp MP						
*Pav*Ve013	59 M 82 bp PE	389	30,917	66	297,710	6,165,792	5136
	43 M 40 bp MP						
*Pav*Ve037	35 M 82 bp PE	220	61,365	61	263,756	6,050,967	5078
	45 M 40 bp MP						

1. PE: paired-end (ca. 200 bp insert). MP: mate-pair (3–5 kb insert).

2. Millions of reads.

**Figure 2:**
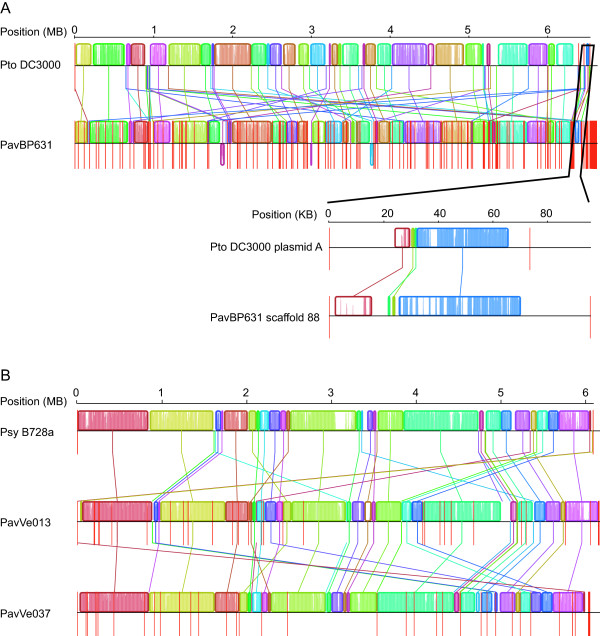
**Whole-genome alignments of*Pav*scaffolds to the most closely related reference sequences.A**. *Pav*BP631 contigs aligned to *Pto* DC3000 reference sequence. Inset: Alignment of scaffold 88 to plasmid A from *Pto* DC3000 (this was done as a separate analysis). **B**. *Pav* Ve013 and *Pav* Ve037 contigs aligned to *Psy* B728a reference sequence. Each colored block represents a local colinearity block that can be aligned between strains without any rearrangements. White spaces within blocks indicate regions of low sequence conservation. Vertical red lines indicate scaffold breaks for *Pav* sequences or boundaries between chromosomes/plasmids in the case of the *Pto* DC3000 reference sequence. Alignments were generated using progressiveMauve [20].

When the contigs were scaffolded using 38–45 million mate-pairs, the N50 improved to 79 kb for *Pav* BP631 and to 264–298 kb for the other strains (Table 1). The total genome sizes were 6.6 megabases (Mb) for *Pav* BP631 and 6.1 to 6.2 Mb for the other two strains, consistent with the presence of extra-chromosomal plasmids in *Pav* BP631. *Pav* Ve013 and *Pav* Ve037 are largely colinear with the phylogroup 2 reference strain *Psy* B728a, while *Pav* BP631 displays substantially more rearrangement relative to *Pto* DC3000, the reference strain for phylogroup 1 (Figure 2). There is a 95 kb scaffold in *Pav* BP631 that is made up of high-coverage contigs and is colinear with plasmid A from *Pto* DC3000 over about half of its length.

### Ortholog analysis

**Figure 3:**
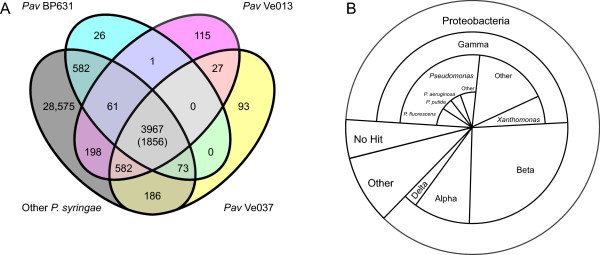
**A. Overlap of ortholog groups between*Pav*strains and 24 other*P. syringae*strains.** Numbers inside Venn diagram indicate the number of ortholog groups with ORFs in each of the strains represented. The number in brackets in the central cell indicates the number of ortholog groups with at least one representative in each *P. syringae* strain (core genes). B. Phylogenetic distribution of top BLAST hits of *Pav* genes with no orthologs in non*-Pav P. syringae* strains.

The RAST annotation sever predicted between 4816 and 5136 open reading frames (ORFs) per strain (Table 1) which were grouped into between 4710 and 4951 ortholog groups by orthoMCL (Figure 3a). There were 3967 ortholog families shared among the three *Pav* strains, all of which were also found in other strains. Of these, 1856 were found in all 29 *P. syringae* strains, comprising the operational *P. syringae* core genome. Each *Pav* strain had between 26 and 115 unique genes that lack orthologs in any other *P. syringae* strain. The closely related *Pav* Ve013 and *Pav* Ve037 strains shared 27 ORFs that lacked orthologs in any other *P. syringae* strain, while there were no ORFs found only in the three *Pav* strains and no other *P. syringae* strain.

There were a total of 262 Pav- specific homology groups that lacked orthologs in any other Psy strain in the ortholog analysis section of the results. Approximately half of these were most similar to genes from other species in the gamma-Proteobacteria, while another 25% were most similar to genes from beta-Proteobacterial species (Figure 3b). Over half of the ORFs with gamma-Proteobacterial hits matched genes from other *Pseudomonas* species, while ~15% were to genes from the plant pathogen *Xanthomonas campestris*. Of the 142 *Pav*-specific genes in *Pav* Ve013, 101 were located in two large gene clusters. One of these was a 110 kb cluster of 43 genes inserted at a tRNA locus in a region that is syntenic between *Pav* Ve013 and *Psy* B728a (Additional file 1: Figure S1). Of these genes, 32 are most similar to *Xanthomonas campestris* 8004 genes (>50% overlap; E-value <10^-10^), including a type IV secretion gene and a transposase gene located at one end of the cluster. The second cluster is 175 kb in length and consists of 58 genes, including 17 that are shared with *Pav* Ve037 (Additional file 2: Figure S2). The central core of this region comprises a 49 kb PFGI-1 type integrative conjugative element (ICE), most of which is homologous to an ICE from *Pseudomonas fluorescens* SWB25.

### Recombination and phylogenetic analysis

Comparisons of genealogies for each gene greater than 300 bp in length to the genome tree identified seven putatively recombinant genes where *Pav* BP631 is sister to one or both of the other *Pav* strains. However, in two cases all but one of the sequences are from *Pav* strains, so *Pav* BP631 necessarily has to be sister to other *Pav* strains in the unrooted tree. Three of the remaining five have very poor branch support. The remaining two putatively recombinant genes, a GAD-like protein and a putative prophage lysozyme, cluster *Pav* BP631 with one of the other *Pav* strains, but not both. In both cases the gene trees are highly incongruent with the core genome phylogeny, so it is not possible to determine the direction of transfer. Indeed, there are relatively long terminal branches leading to the *Pav* strains, suggesting that both *Pav* strains horizontally acquired the gene from other un-sequenced strains or that their relation may be an artifact of long-branch attraction.

**Figure 4:**
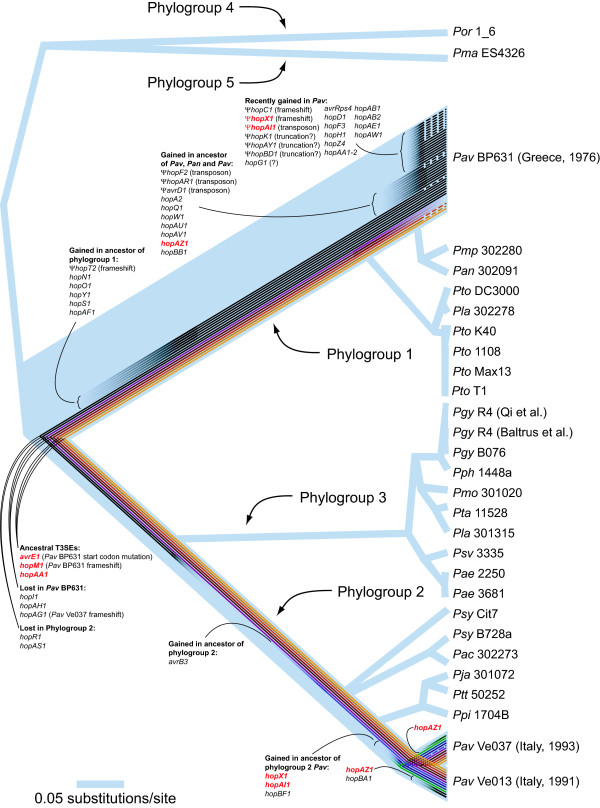
**Whole-genome phylogenetic relationships among*P. syringae*strains with evolutionary histories of*Pav*T3SEs mapped onto branches.** Each line within the branches represents one T3SE and indicates when it was acquired or lost by the ancestors of the *Pav* strains. Dashed lines indicate that a T3SE has become a pseudogene. T3SEs that are present in all *Pav* strains are indicated in red. Lines representing T3SEs in phylogroup 2 are arbitrarily colored to aid in following them between strains. Phylogroup designations follow [1]. All branches have 100% aLRT support except for the relationships among *Pto* strains K40, 1108, Max13 and T1.

When 42,569 variable positions from 595 single-copy orthologous genes in each of the 29 genome sequences were used for phylogenetic analysis the relationships were consistent with previous MLSA studies, although with much stronger phylogenetic support (Figure 4). There was 100% approximate Likelihood Ratio Test (aLRT) support for every node except for two of the relationships within the *Pto* lineage. In phylogroup 1, *Pav* BP631 clustered with *Pan* 302091 and *Pmo* 301020, sister to five *Pto* strains and *Pla* 302278. In phylogroup 2, *Pav* Ve013 and *Pav* Ve037 cluster as a sister lineage to *Pja*, 301072, *Ptt* 50252 and *Ppi* 1704B within a group that also included *Psy* Cit7, *Pac* 302273 and *Psy* B728a. These two phylogroups clustered with the phylogroup 3 lineage that included 10 of the twelve additional sequenced strains, to the exclusion of the single representatives of phylogroups 4 and 5. The rooting of the tree is uncertain since the phylogenetic analysis did not include outgroups.

### Divergence times

Divergence time estimates were strongly dependent on the substitution rate priors specified (Table 2). Using the slower rate based on the divergence of *E. coli* from *Salmonella* 140 million years ago, we obtained age estimates for the most recent common ancestor of all *P. syringae* isolates ranging from 150 to 183 million years, depending on the locus. Phylogroup 1 *Pav* strains are inferred to have diverged between 3 and 10 million years ago, while phylogroup 2 strains have ages ranging from 17 to 34 million. When the substitution rate is inferred from the emergence of a clonal lineage of methicillin-resistant *Staphylococcus aureus* (MRSA) since 1990 [21], *P. syringae* is inferred to have diversified within the last 42,000 to 74,000 years. Even with this rapid rate the data are not consistent with emergence of *Pav* within the last 40 years as the minimum age within the 95% confidence interval of any of the loci is 281 years for phylogroup 1 *Pav* and 2210 years for phylogroup 2 *Pav.* Phylogroup 2 *Pav* is inferred to have emerged thousands of years before phylogroup 1 *Pav* (4500–12,000 years versus 1200–1700 years).

**Table 2:** **Divergence time estimates for*Pav*lineages**

**Calibration point**	**Rate (subst./yr)**	**Locus**	**Age of Most Recent Common Ancestor (mean, 95% CI)**^**1**^		
			***P. syringae***	**Phylogroup 1 *Pav***	**Phylogroup 2 *Pav***
*E. coli-Salmonella* (140 MYA^2^) [22]	1x10^-9^	*gapA*	183 MYA (92.8-300)	3.16 MYA (0.831-6.39)	29.5 MYA (16.9-44.5)
		*gltA*	171MYA (75.4-300)	3.88 MYA (0.945-8.02)	17.6 MYA (7.10-29.8)
		*gyrB*	171 MYA (93.7-272)	10.1 MYA (2.62-19.5)	34.3 MYA (17.9-54.8)
		*rpoD*	153 MYA (66.4-260)	5.23 MYA (1.61-9.80)	14.8 (7.17-23.1)
MRSA (1990) [21]	2x10^-6^	*gapA*	74,000 (39,800-116,000)	1200 (281–2350)	12,000 (7270–17,400)
		*gltA*	41,600 (22,200-67,400)	1380 (414–2690)	4560 (2210–7070)
		*gyrB*	51,900 (30,500-77,700)	3400 (1050–6480)	10,600 (5580–16,700)
		*rpoD*	49,600 (24,400-82,300)	1740 (640–3170)	7270 (3810–11,700)

1. Years before present unless otherwise indicated.

2. Million years before present.

### Type III secreted effectors

There are dramatic differences in the number of T3SE homologs encoded in the genome of *Pav* BP631 versus the two other strains (Figure 4). *Pav* BP631 has homologs of 38 T3SEs, of which five have frameshift mutations and four have transposon insertions. There are partial sequences of three additional T3SEs, suggesting that they are truncated. However, they are located at the ends of scaffolds, so we are unable to confirm this. The entire sequence of a fourth T3SE that is also located at the end of a scaffold, *hopG1*, is present except for the stop codon. In contrast, *Pav* Ve013 and *Pav* Ve037 have homologs of only twelve and eleven T3SEs respectively, and one of these, *hopAG1,* is disrupted by a frameshift in *Pav* Ve037.

Only six T3SE homologs are common to all three *Pav* strains, and four of these are putatively non-functional in *Pav* BP631. Three of these shared T3SEs (*avrE1, hopM1,* and *hopAA1*) are also present in all other *P. syringae* strains and have genealogical histories congruent with the core genome phylogeny of the species, though *hopM1* is truncated in many strains. These three T3SEs are located in the conserved effector locus (CEL) that flanks the type III secretion system structural genes. The *Pav* BP631 *hopM1* locus has a number of frameshift mutations, while the *avrE1* gene contains a mutation in the first codon, changing GTG to GTA, which is a highly-atypical start codon that very likely severely reduces or completely disrupts translation [23]. The only shared and putatively functional T3SE in the CEL is *hopAA1*.

The other T3SE homologs that are present in all three *Pav* strains are *hopAI1*, which is truncated in *Pav* BP631, *hopX1*, which has a frameshift in *Pav* BP631, and *hopAZ1*. All three *Pav* strains carry *hopX1* in the exchangeable effector locus (EEL), which is located on the opposite side of the type III secretion system structural genes as the CEL, and which contains a variable assortment of T3SEs that are flanked by conserved genes. The EEL of *Pav* Ve013 and *Pav* Ve037 also contain *avrB3* while the EEL of *Pav* BP631 contains a *hopF2* sequence that has been disrupted by a transposase. Both *hopX1* and *hopAI1* appear to have been acquired independently by the two *Pav* lineages after their divergence from their most recent non-*Pav* common ancestor. The *hopAZ1* T3SE is particularly interesting since it is intact and putatively functional in all three *Pav* strains, yet appears to have been acquired independently by all three. No *Pav* HopAZ1 sequence shares more than 71% amino acid identity with any other *Pav* sequence, and they each form very strongly supported distinct phylogenetic clusters with other HopAZ1 alleles (Additional file 3: Figure S3).

Five other T3SEs are present in the majority of *P. syringae* strains and have phylogenies congruent with the core genome. These include two that were lost in the common ancestor of all phylogroup 2 strains (*hopR1* and *hopAS1*) and three that have recently been lost in the phylogroup 1 *Pav* lineage (*hopI1*, *hopAH1* and *hopAG1*). All other *Pav* T3SEs have been acquired by horizontal transfer since the two *Pav* lineages diverged from each other. In the phylogroup 2 lineage, *avrB3* was acquired by the common ancestor of all phylogroup 2 strains, *hopBF1* was acquired by the common ancestor of phylogroup 2 *Pav*, and *hopBA1* was acquired by *Pav* Ve013 since its divergence from *Pav* Ve037. In the phylogroup 1 lineage, six T3SEs were acquired by the common ancestor of all phylogroup 1 strains. Nine additional T3SEs (plus *hopAZ1*) were acquired by the common ancestor of *Pav* BP631, *Pmp* 302280 and *Pan* 302191. However, the majority of T3SE gain has occurred since *Pav* BP631 diverged from its common ancestor with *Pmp* 302280 and *Pan* 302191 (15, plus *hopX1* and *hopAI1*), almost half of which are pseudogenes.

## Discussion

The hazelnut decline pathogen *P. syringae* pv. *avellanae* provides a striking example of convergent evolution of host-specificity. While both *Pav* lineages are part of the *P. syringae* species complex, one must go back to the origin of the species complex to find their most recent common ancestor [6]. The fact that these two lineages began causing disease on hazelnut at roughly the same time and give rise to similar disease phenotypes makes it seem unlikely that their convergent evolution occurred entirely independently. However, we find almost no evidence of genetic exchange between these lineages, and little similarity in their respective virulence gene complements.

Hazelnut decline was first described in Greece caused by phylogroup 1 *Pav*, yet there is strong evidence that phylogroup 2 *Pav* emerged first*.* MLSA studies show that the phylogroup 2 *Pav* clade, which is restricted to Italian isolates, has over four times the genetic diversity found among the phylogroup 1 *Pav* strains, which include both Greek and Italian isolates [6]. This is significant since the extent of genetic diversity is usually associated with evolutionary age (baring the influence of certain evolutionary process or demographic changes). This is borne out by our molecular dating results. There is large variation in absolute divergence times depending on the substitution rate used, as rates based on fossil evidence [22] are several orders of magnitude higher than rates based on emergence of antibiotic resistant bacteria [21], diversification within hosts [21,24], or ancient DNA [25]. Despite these limitations, one clear point is that divergence times are three to ten times older for phylogroup 2 *Pav* than for phylogroup 1 *Pav*. Indeed, even the most rapid substitution rates result in estimated divergence times for both lineages that predate the emergence of hazelnut decline by thousands of years.

The finding that *Pav* has been diversifying for a long period of time without being observed in the field is surprising. In Greece, *Pav* had a particularly heavy impact on the hazelnut cultivar Palaz during the late 1970s [3]. This cultivar was introduced from Turkey in the late 1960s where there are no records of hazelnut bacterial canker. In contrast, there has been a long history of hazelnut cultivation in Italy, although the Palaz cultivar is not grown. Italian hazelnut cultivation increased rapidly during the decades leading up to the first observed outbreak during the 1970s, going from 3500 hectares in 1945 to almost 20,000 hectares by 1990 in the province of Viterbo [26]. Much of the new cultivation in both Greece and Italy occurred on marginal lands with acidic soils, which are conditions that are likely to make hazelnut more susceptible to *Pav* infection.

How can the long time since *Pav* divergence be reconciled with the recent occurrence of hazelnut decline? Microbiological surveys of in Italy have found that wild hazelnut trees are often infected by phylogroup 2 *Pav*[27], suggesting that wild trees might act as a reservoir. It is possible that phylogroup 1 *Pav* are associated with wild hazelnut in Greece, but similar surveys have not been carried out. Taken together, these data strongly suggest that both *Pav* lineages have been cryptically infecting hazelnut trees or wild relatives for a long time, and that the emergence of hazelnut decline in the 1970s was most probably due to changes in agricultural practice.

While there is no evidence of horizontal transfer between *Pav* lineages, we do find a large number of genes that have been horizontally acquired from other bacteria. Over 250 ORFs from the three *Pav* genomes lack orthologs in any other sequenced *P. syringae* strain. This includes over 200 genes that are present in one of the phylogroup 2 *Pav* strains but not the other, suggesting extensive gene acquisition and loss in this lineage. Over 80% of these genes have homologs in other Proteobacteria. Many of the strain-specific genes are organized into large genomic islands with signatures of mobile elements. Two of these genomic islands are homologous to regions found in other plant-associated bacteria, although the genetic similarity is low. This suggests either that the genetic exchange occurred in the distant past or that the donor strain is only distantly related to the sequenced strains in the database. It would be interesting to sequence other hazelnut-associated bacteria such *Xanthomonas arbicola* pv. *corylina*, which is responsible for hazelnut blight and *Pseudomonas fluorescens* strains associated with the roots of hazelnut trees.

A remarkable feature of evolution of phylogroup 1 *Pav* is the extremely fluid nature of their T3SE repertoires. Like other phylogroup 1 strains, the frequency of T3SE acquisition is extremely high, with 27 T3SEs acquired since it diverged from the common ancestor of the group. However, the rate of T3SE loss is much higher than has been documented for any other *P. syringae* strain. A total of twelve *Pav* BP631 T3SEs are inferred to be non-functional. By comparison, the strain with the second most T3SE pseudogenes is *Pto* DC3000 with seven [16]. All of the pseudogenization events in *Pav* BP631 appear to have happened since it diverged from *Pmp* 302280 and *Pan* 302091. Indeed, seven of them involve T3SEs that were acquired since this divergence, meaning that they were either acquired as nonfunctional genes or that they became pseudogenes after acquisition. The frequency of T3SE gain and loss is much lower in the phylogroup 2 *Pav* strains, with six and five gains for *Pav* Ve013 and *Pav* Ve037 respectively since they diverged from other phylogroup 2 strains. This is typical of the phylogroup as a whole, with three other strains that have acquired six or less T3SEs and the largest number of T3SE gains being twelve in *Ppi* 1704B.

Two of the *Pav* BP631 T3SE putative pseudogenes, *avrE1* and *hopM1,* are notable because they are located in the CEL, which is present in all *P. syringae* strains with canonical *hrp*/*hrc* type III secretion systems. AvrE1 is essential for virulence in some *P. syringae* strains [28], but is functionally redundant with HopM1 in *Pto* DC3000, where it suppresses salicylic acid-mediated immunity [29]. Frameshift mutations and truncations are common in *hopM1*, including in *Pph* 1448A [8], *P. syringae* pv. *aptata* DSM 50252 [4] and *Pto* T1 [10]. To date, all sequenced strains have had intact *avrE1* genes, except for *Psv* 3335 [15], which has a contig break in the gene and *Por* 1_6, which has a premature stop codon, but has an intact *hopM1* gene [14]. Homologs of *avrE* are also present in a number of other plant pathogens, including *Erwinia amylovora* and *Pantoea stewartii*, where it is essential for virulence [30-32]. Since *P. syringae* mutants lacking both of these T3SEs have strongly impaired virulence [33] it is unclear how *Pav* BP631 is able to establish infection without functional copies of either gene. It is possible that HopR1 [34] or another uncharacterized T3SE compensate for the loss of AvrE and HopM1 in hazelnut. Alternatively, a low level of translation might be initiated off the highly-atypical GTA start codon in *avrE*[23] or another in-frame start codon might be used, though this would be likely to have drastic effects on the N-terminal secretion signal and there are no other obvious candidates for ribosome binding sites.

Of the twelve putatively non-functional T3SEs in *Pav* BP631, four have intact homologs in phylogroup 2 *Pav.* These include the two CEL T3SEs discussed above and two T3SEs (*hopX1* and *hopAI1*) that were independently acquired in each *Pav* lineage since they diverged from their closest sequenced relatives. Furthermore, three additional T3SEs that are present in phylogroup 2 *Pav* are inferred to have been lost completely in *Pav* BP631 since it's divergence from *Pmp* and *Pan.* This striking pattern suggests that phylogroup 1 *Pav* BP631 was under strong selective pressure to lose T3SEs deployed by the other *Pav* lineage.

The only putatively functional T3SEs that are common among the three *Pav* strains are HopAA1 and HopAZ1. HopAA1 is encoded in the CEL and descended from the common ancestor of *P. syringae*. It has been shown to play a role in the suppression of innate immunity in Arabidopsis [35]. *Pav* BP631 also carries a paralogous copy (in-paralog) of *hopAA1* in addition to the one in the CEL. This paralogous *hopAA1* allele is also present in the two strong Arabidopsis pathogens *Pto* DC3000 and *Pma* ES4326. One of the most interesting findings is that *hopAZ1* was independently acquired in all three *Pav* strains, which points to HopAZ1 as a promising candidate for modulating hazelnut host specificity. Unfortunately, this T3SE has not been functionally characterized and has no conserved domains. HopAZ1 alleles are present in twelve of the 29 *P. syringae* strains with sequenced genomes and dispersed among four of five phylogroups. A genealogical analysis of the *hopAZ1* family shows strong discordance from the evolutionary history of the core genome, indicating frequent horizontal transmission of this T3SE family (Additional file 3: Figure S3).

## Conclusions

Our comparative genomic analysis of three *Pav* isolates has further confirmed convergent evolution of two independent lineages onto hazelnut, and that this convergence is not due to genetic exchange between lineages. Furthermore, the divergence in T3SE complements suggests that the molecular mechanisms of defense evasion are distinct in each lineage. There has been particularly extensive remodeling of its T3SE repertoire in the more recently emerged lineage possibly in response to recognition by host factors that have coevolved with the T3SEs deployed by the other lineage. However, both lineages have been diversifying as hazelnut pathogens since long before the initial hazelnut decline outbreak was first documented in 1976. This suggests that changes in agricultural practice such as the propagation of new cultivars in Greece in the 1960s and 70s and the expansion of hazelnut cultivation into marginal habitats in Italy may have provided suitable conditions for the epidemic emergence of previously cryptic pathogens. While this scenario is clearly conjecture, we now have a number of strong candidate loci to pursue. Functional characterization of these loci in the future may reveal the key steps that these two distinct lineages took in order to subvert the hazelnut immune system.

## Methods

Sequencing and genome assembly followed the methods described in [36]. Briefly, cells were harvested from 1 mL of stationary-phase culture and DNA was isolated using the Gram-negative bacterial culture protocol of the Puregene Genomic DNA Purification Kit (Qiagen Canada, Toronto, ON) using double volumes of each reagent, repeating the protein precipitation step twice, and spooling the DNA during the precipitation step. Paired-end and mate-pair sequencing libraries were prepared using sample preparation kits from Illumina (San Diego, CA). DNA was sheared to 200 base pairs (bp) for the paired-end libraries and to 3 kilobases (kb) for the mate-pair libraries using a Covaris S-series sample preparation system. Each library was run on a single lane of an Illumina GA IIx sequencer, for 38 cycles per end, except for the *Pav* Ve013 and *Pav* Ve037 paired-end libraries, which were run for 82 cycles per end. Paired-end reads were assembled using the CLC Genomics Workbench (Århus, Denmark), using the short-read de novo assembler for *Pav* BP631 and the long-read assembler for the other strains. The resultant contigs were scaffolded with the mate-pair data using SSPACE [37]. Scaffolds were ordered and oriented relative to the most closely related fully sequenced genome sequence (*Pto* DC3000 for *Pav*BP631; *Psy* B728a for the other strains) using the contig mover tool in Mauve [20]. Automated gene prediction and annotation was carried out using the RAST annotation server [38]. These Whole Genome Shotgun projects have been deposited at DDBJ/EMBL/GenBank under the accession numbers AKBS00000000 (*Pav* BP631), AKCJ00000000 (*Pav* Ve013) and AKCK00000000 (*Pav* Ve037). The versions described in this paper are the first versions, AKBS01000000, AKCJ01000000 and AKCK01000000. Our methods have been shown to correctly assemble >95% of the coding sequences, including >98% of single-copy genes for the fully sequenced strain *P. syringae* pv. *phaseolicola* (*Pph*) 1448A [36].

The amino acid translations of the predicted ORFs from each strain were compared to each other and to those from 26 other publically available *P. syringae* genome sequences using BLAST [39] and were grouped into orthologous gene families using orthoMCL [40]. *Pav* ORFs that were less than 300 bp in length and that did not have orthologs in any other strain were excluded from further analyses. The DNA sequences of the remaining *Pav*-specific ORFs were compared to all other strains using BLASTn and those that matched over at least 50% of their length with an E-value < 10^-20^ were also excluded. The amino acid translations of the remaining *Pav*-specific genes were searched against GenBank using BLASTp to determine putative functions and the taxonomic identities of donor strains. Genomic scaffolds containing blocks of *Pav*-specific genes were compared to the genome sequences of the most closely related *Pav* reference strain and to the database strain with the most hits to ORFs in the cluster using BLASTn and similarities were visualized using the Artemis Comparison Tool [41].

Amino acid sequences of ortholog groups were aligned using MUSCLE [42], and back-translated to DNA alignments using TranslatorX [43]. PhyML [44] was used to infer phylogenies for each ortholog group and phylogenetic confidence was determined by the approximate likelihood-ratio test for branches (aLRT) method [45]. PhyML was also used to infer the core genome phylogeny by concatenating the aligned sequences of each ortholog group with one representative sequence in each strain and removing conserved alignment positions. Recombination between *Pav* lineages was detected by identifying gene trees in which *Pav* BP631 formed a monophyletic group with one or both of the other *Pav* strains.

In addition to the whole-genome ortholog analysis, we identified T3SE pseudogenes and gene fragments by BLASTing all of the amino acid sequences of T3SEs in the database at http://www.pseudomonas-syringae.org against the *Pav* genome sequences, as well as 24 other draft *Psy* genome sequences using tBLASTn. Homologous DNA sequences were extracted and examined for truncations, frameshifts, contig breaks (usually caused by the presence of transposases or other multi-copy elements disrupting the coding sequences), and chimeric proteins. Sanger sequencing was used to fill contig gaps in *Pav* T3SE orthologs and to confirm frameshift mutations and transposon insertions using primers flanking each gap. Sequences lacking frameshifts were translated to amino acid sequences, aligned using MUSCLE, and back-translated to DNA alignments using TranslatorX [43]. Sequences with frameshifts were added to the nucleotide alignments using MAFFT [46]. Phylogenies were inferred for each alignment using PhyML. Gains and loss of each T3SE family was mapped onto the core genome phylogeny by identifying clades in each T3SE gene tree that are congruent with the core genome phylogeny, allowing for gene loss in some lineages.

Divergence times were estimated for the most recent common ancestor of each of the *Pav* lineages and for *P. syringae* as a whole using the MLSA dataset from Wang *et al.*[6]. This included partial sequences of four protein-coding genes for ten phylogroup 1 *Pav* strains and twelve phylogroup 2 *Pav* strains, as well as 110 additional *P. syringae* strains. Analyses were carried out using an uncorrelated lognormal relaxed molecular clock in BEAST v1.6.2 [47] with unlinked trees, and substitution models, allowing for recombination between loci. The HKY substitution model was used with gamma-distributed rate variation, with separate partitions for codon positions 1 + 2 and for third positions. Substitution rates were set to published rates based on the split of *Escherichia coli* and *Salmonella*[22] and the emergence of methicillin resistant *Staphylococcus aureus* (MRSA) [21]. Two independent Markov chains were run for 50 Million generations and results were combined for parameter estimates.

## Abbreviations

CEL, Conserved effector locus; EEL, Exchangeable effector locus; MLSA, Multilocus sequence analysis; MYA, Millions of years ago; T3SE, Type III secreted effector; Bp, Base pairs; Kb, Kilobases; Pan, P. syringae pv. actinidiae; Pav, Pseudomonas syringae pv. avellanae; Pja, P. syringae pv. japonica; Pmp, P. syringae pv. morsprunorum; Ppi, P. syringae pv. pisi; Psy, P. syringae pv. syringae; Pto, P. syringae pv. tomato; Ptt, P. syringae pv. aptata.

## Competing interest

The authors declare no competing interests.

## Authors’ contributions

DSG, HEOB and MS conceived and designed the experiments. CY, PF, LY, JZ and PWW performed the experiments. HEOB, ST and YG analyzed the data. DSG Contributed reagents and materials. DSG, HEOB and MS wrote the paper. All authors read and approved the final manuscript.

## Supplementary Material

Additional file 1 **Figure S1.BLASTn-based comparison of*Pav*Ve013*, Psy*B728a and*Xanthomonas campestris*8004 showing a 110 kb insertion in*Pav*Ve013 with portions that are homologous to three different regions in the*X. campestris*8004 genome.** Click here for file

Additional file 2 **Figure S2.BLASTn-based comparison of*Pav*Ve013*, Pav*Ve037*, Psy*B728a and*Pseudomonas fluorescens*SBW25 showing large insertions in both*Pav*strains which lack homology to each other except for a central core homologous to an integrative conjugative element (ICE) in*P. fluorescens*SBW25.** Click here for file

Additional file 3 **Figure S3.Gene tree for*hopAZ*homologs from all sequenced*P. syringae*strains.*Pav*sequences, which are colored in red, are found in three major subclades.** Numbers above branches indicate aLRT branch support values. Click here for file
