# Supplementary figures and images for: Extensive remodeling of the Pseudomonas syringae pv. avellanae type III secretome associated with two independent host shifts onto hazelnut
Source: BMC Microbiol. 2012 Jul 16;12:141. doi: 10.1186/1471-2180-12-141 (PMC3411506; doi:10.1186/1471-2180-12-141)

Psy B728a

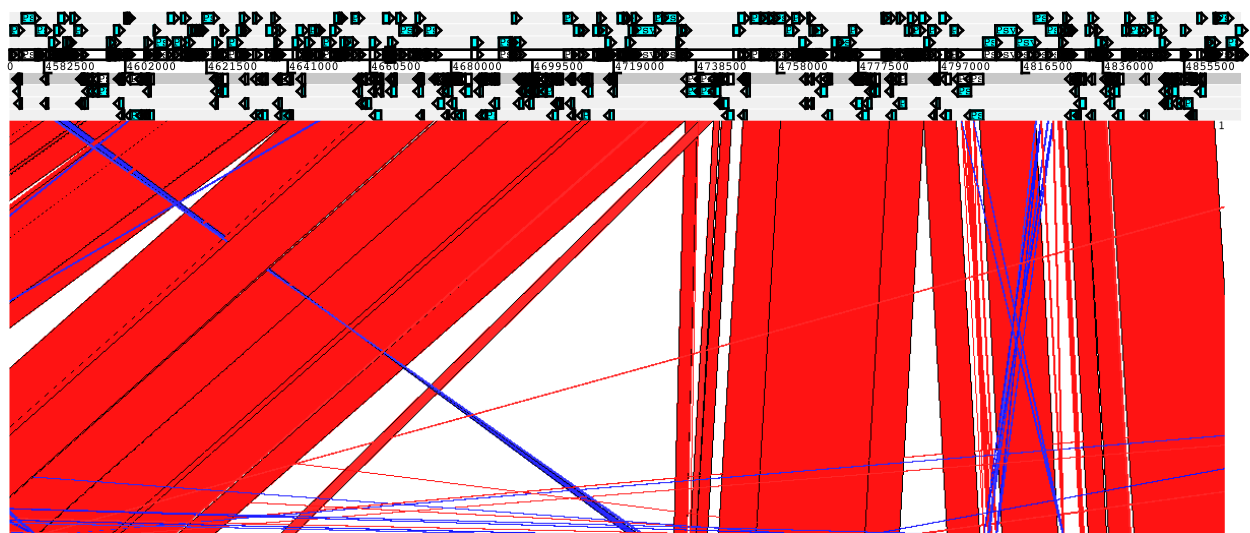

Pav 013

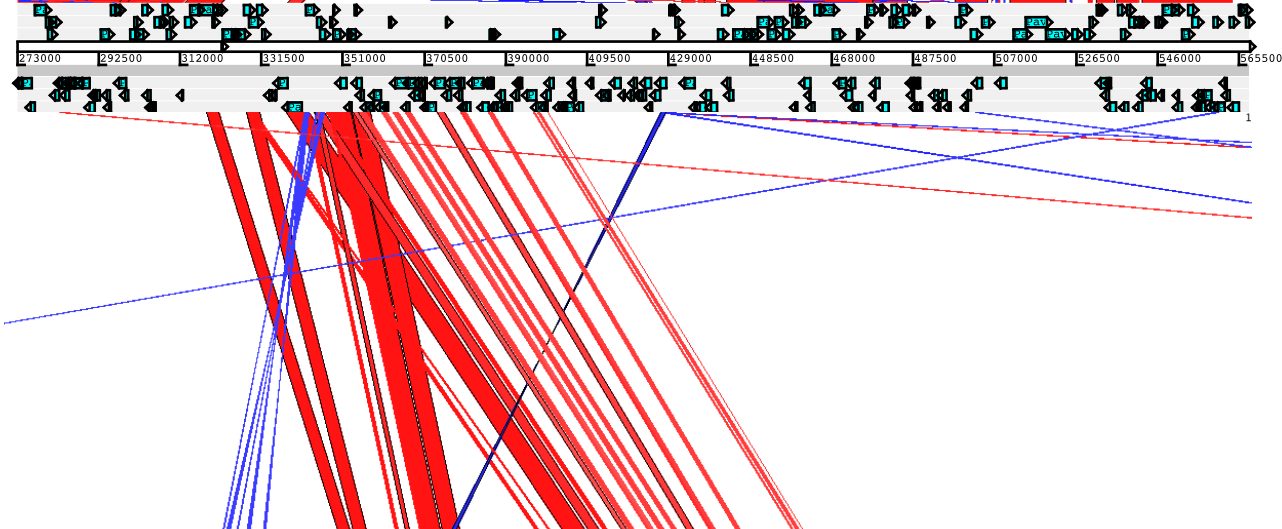

*X. campestris*  
8004

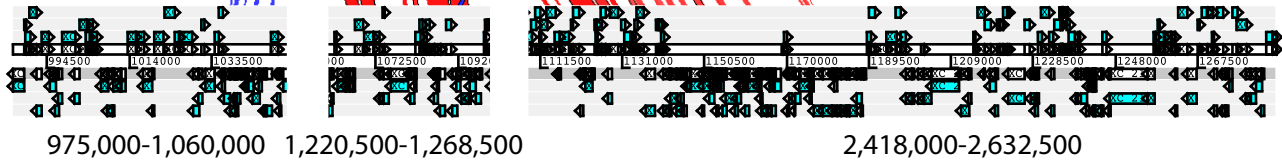

Supplement: Additional file 1 — Figure S1.BLASTn-based comparison ofPavVe013, PsyB728a andXanthomonas campestris8004 showing a 110 kb insertion inPavVe013 with portions that are homologous to three different regions in theX. campestris8004 genome. [file 1471-2180-12-141-S1.pdf]

Psy B728a

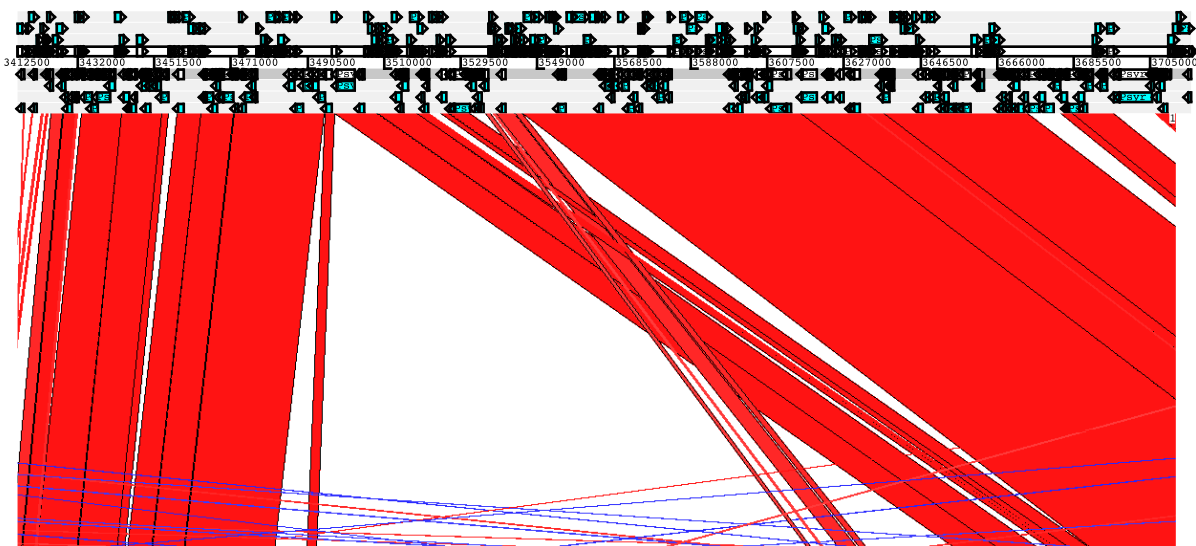

Pav 013

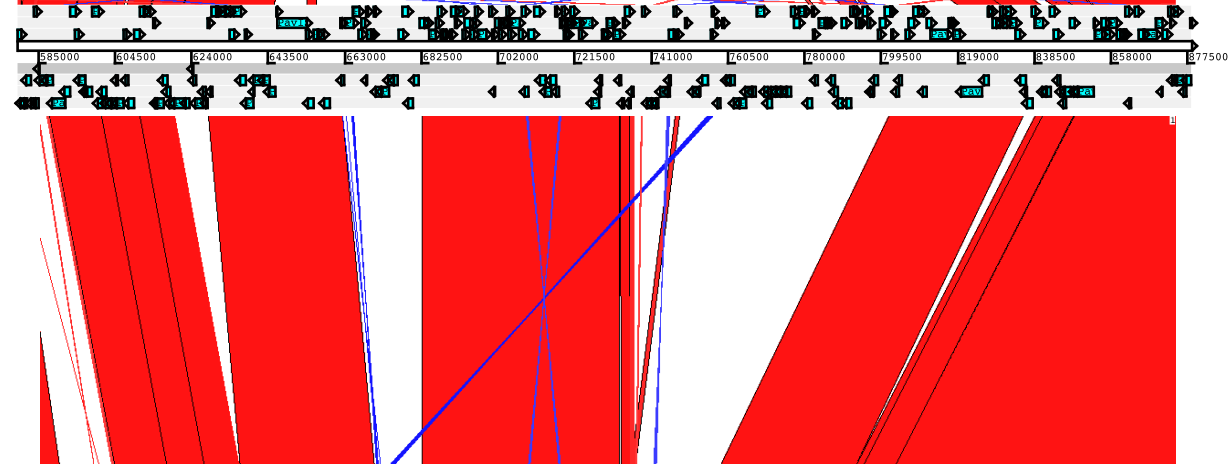

Pav 037

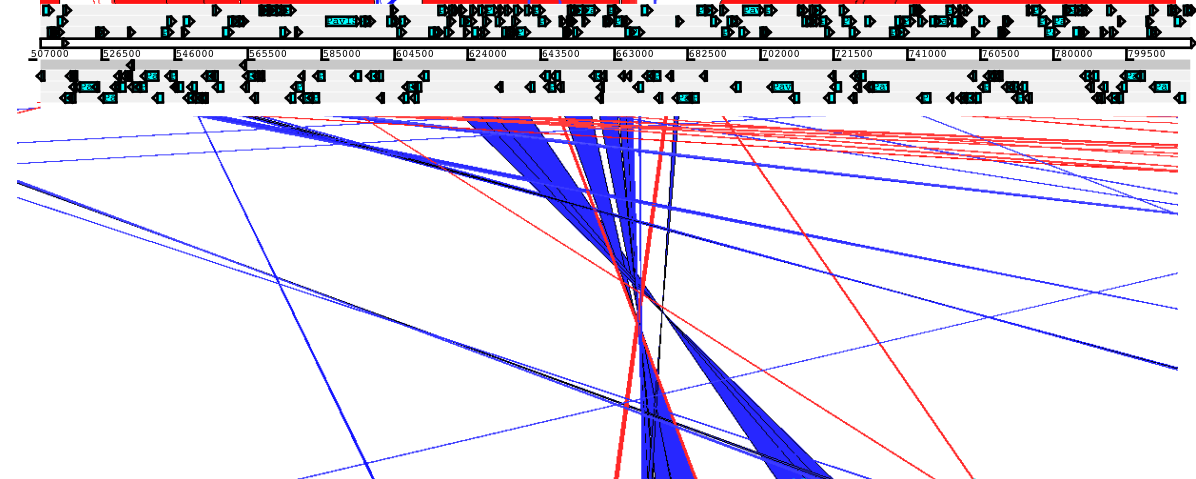

*P. fluorescens*  
SBW25

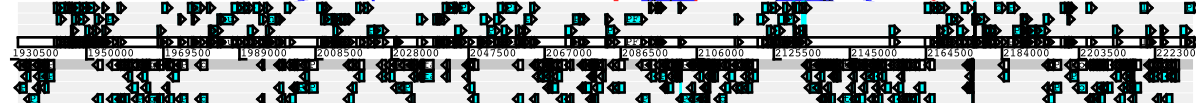

Supplement: Additional file 2 — Figure S2.BLASTn-based comparison ofPavVe013, PavVe037, PsyB728a andPseudomonas fluorescensSBW25 showing large insertions in bothPavstrains which lack homology to each other except for a central core homologous to an integrative conjugative element (ICE) inP. fluorescensSBW25. [file 1471-2180-12-141-S2.pdf]

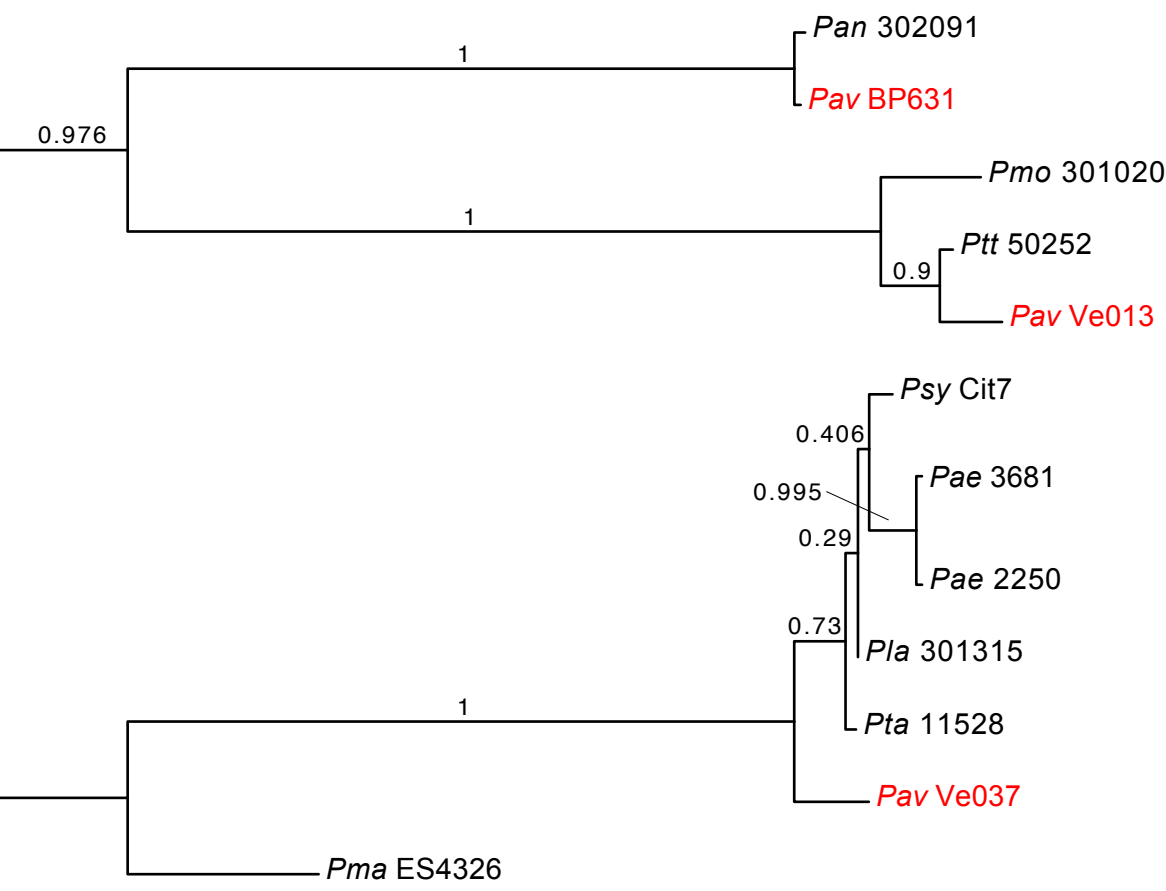

0.05 substitutions/site

Supplement: Additional file 3 — Figure S3.Gene tree forhopAZhomologs from all sequencedP. syringaestrains.Pavsequences, which are colored in red, are found in three major subclades. Numbers above branches indicate aLRT branch support values. [file 1471-2180-12-141-S3.pdf]
